# Active and Passive Euthanasia: Current Opinion of Mexican Medical Students

**DOI:** 10.7759/cureus.3047

**Published:** 2018-07-25

**Authors:** Alejandro Gutierrez Castillo, Javier Gutierrez Castillo

**Affiliations:** 1 Escuela De Medicina, Instituto Tecnológico Y De Estudios Superiores De Monterrey, Nuevo León, MEX; 2 Escuela De Medicina, Instituto Tecnológico Y De Estudios Superiores De Monterrey, Nuevo Leon, MEX

**Keywords:** euthanasia, medical student, physician assisted death, bioethics, clinical ethics, religion, mexico

## Abstract

Background: The idea to accelerate the process of death in a terminally ill patient is an issue that has polarized societies since ancient times. The purpose of this study is to describe and analyze the opinion of medical students from Nuevo Leon, Mexico, about passive euthanasia, active euthanasia, and their personal posture on the topic.

Material and Methods: Using a three-part questionnaire, 1,319 medical students of the first three years of medical school, from three of the four medical schools in the state, were interviewed. The questionnaire included questions on demographics, religion, and the personal posture of the student on active euthanasia, passive euthanasia, and their personal posture on the topic.

Results: Of those interviewed, 44.4% were in favor of active euthanasia, 52.1% of passive euthanasia, and 44.8% had a positive personal posture on the topic. Age and grade were not significant variables for the posture of the students, but the variable gender showed a predominantly positive posture in the male subgroup for active (p=0.001) and passive euthanasia (p=0.031). Religion and the importance of religion/spirituality in daily life were the most significant factors (p<0.005) for the interviewees to hold a negative posture in each of the three scenarios. The legal nature of the scenario (p=0.000) and respect for patient's autonomy (p=0.000) were the most important arguments that could change an original negative posture into a positive one.

## Introduction

Etymologically, the word euthanasia comes from the Greek, Eu (good) and Thanatos (death), which means “good death” [1]. The idea of accelerating the process of death in a terminally ill patient is an issue that has been discussed since ancient times. Plato, in Laws, suggested that doctors should be punished by death if by administering any sort of drug, they contribute to the termination of life [2]. While Seneca mentioned that suicide is preferable to a meaningless life and suffering [3]. In contrast, Hippocrates censors it under the Hippocratic oath, where he mentioned, “I will not give poison to anyone though asked to do so, nor I will suggest such a plan” [3]. However, it’s not until the middle ages when this debate headed towards censorship from the perspective of Christian religious beliefs since a person “cannot freely take off his life that was given by a superior entity” [3].

The term euthanasia can be further classified according to the patient's situation and the role of the physician in the process. In passive euthanasia, the role of the physician is limited to suspend treatment or stop extraordinary measures in order to prevent the prolongation of life, restricting to see how the disease process ends with the patient’s life; on the other hand, in active euthanasia, the physician has an active role in ending the patient’s life by administering a toxic substance that accelerates the death of the patient [4]. The relevance of this classification lies in their moral acceptance. For example, according to the American Medical Association (AMA), active euthanasia is strictly forbidden under all circumstances, as it’s incompatible with the physician role as healer, would be difficult or impossible to control, and would pose serious social risks [5]. However, this same institution validates passive euthanasia, as it respects the principle of patient's autonomy only in a previously informed patient with a terminally ill disease [5].

In Mexico, there is still debate about the topic. First, publications showed a low acceptance of euthanasia since only 30% of the surveyed were in favor of passive euthanasia and 18% of its active form [6]. In contrast, a more recent research performed in 2008 showed a positive posture for both cases in 40% of the interviewed people [6]. The purpose of this paper is to describe the attitudes of the medical students from the first three years towards active and passive euthanasia, and their relations according to their demographics.

## Materials and methods

Medical students were surveyed, using the stratified sampling technique, for an observational, retrospective, transversal, analytic study. The sample was divided into three major groups that contained three subgroups each, for a total of nine subgroups. In the first place, the major groups were the school of origin of the participants; the study took into account three out of four medical schools located in Nuevo Leon, Mexico. The year in which the student was enrolled defined the subgroups, only considering the first three years for this study. It is important to mention that the variable “school of origin” was only used for a correct stratified sample. To keep the privacy of the schools, no further analysis of this variable is described. The total distribution of the sample is observed in Table 1.

**Table 1:** Total distribution of the sample according to the school of origin and academic year

		Grade			
		First Year	Second Year	Third Year	Total
School	School 1	59	56	61	176
	School 2	303	304	306	913
	School 3	98	82	50	230
Total		460	442	417	1319

**Figure 1:**
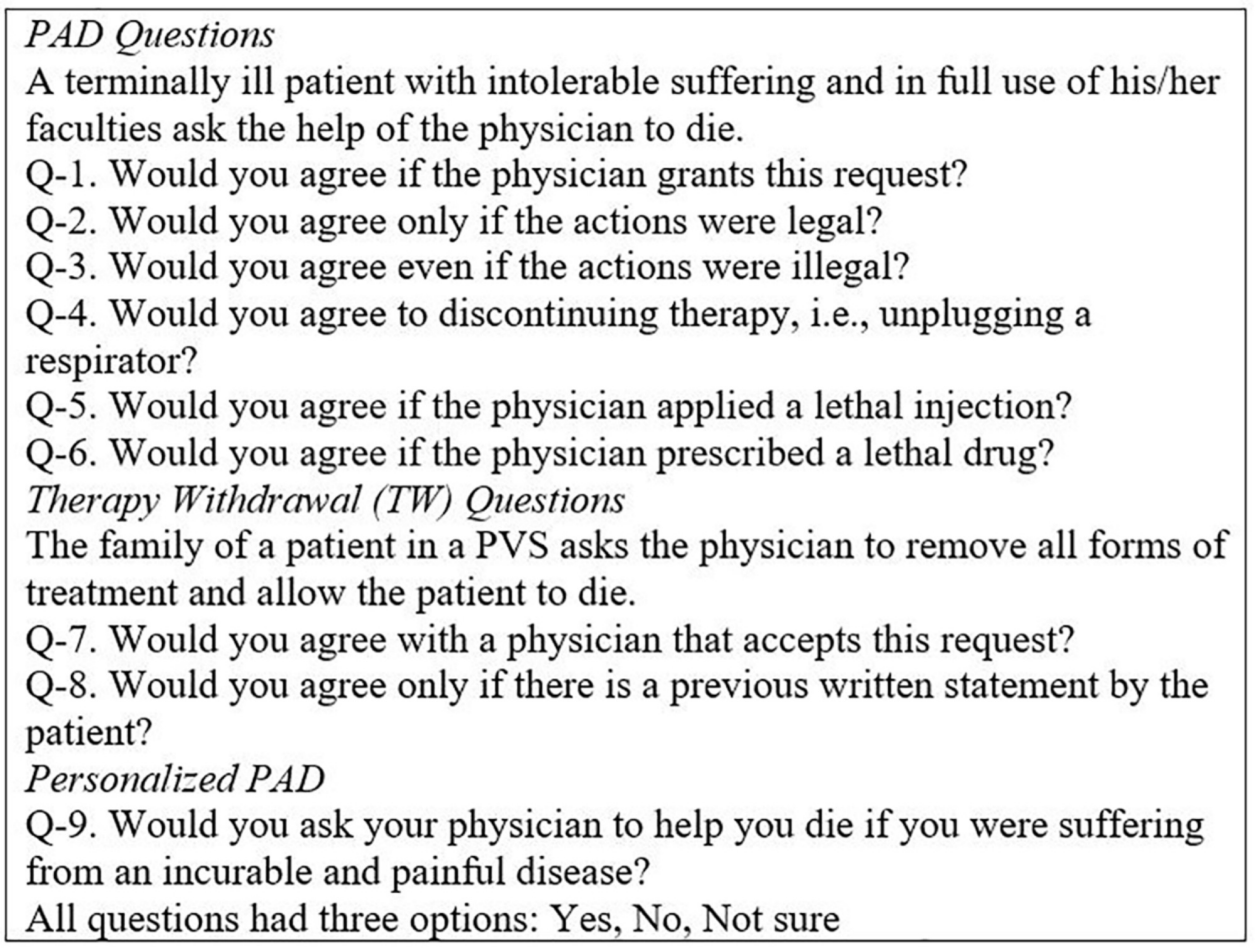
Questionnaire applied to the participants PAD: physician-assisted death; PVS: persistent vegetative state

The questionnaire given to the participants was a modified version of a previously validated tool for other studies [6-7] and was distributed in three parts. The first part was a single question where the student had to agree to be included in the study; only positive answers were included in the study. The second part included five questions that described the demographic information of the students and their religious preferences. The questions were: age (17-30), gender (male or female), grade (first, second, or third year), religious beliefs (Catholic, non-Catholic, no religion), importance of religious beliefs/spirituality in daily life (important, less important, no religion). We decided to classify religion as Catholic and non-Catholic since previous research showed that most of the Mexican population identify themselves as Catholic and their religion has major importance in their daily life [8]. Also, for a better analysis, age was later subdivided into three groups (17-22,23-25,25-30). Finally, the third part of the questionnaire included nine questions that addressed the posture of the surveyed towards passive and active euthanasia and their personal posture to the topic. Questions one, seven, and nine were considered as the main questions that addressed a direct posture for active euthanasia, passive euthanasia, and personal posture towards the topic. Questions two through six were complementary to question one in order to identify specific circumstances that would favor a positive posture towards active euthanasia. Question eight was complementary to question seven, for a specific circumstance that would favor a positive posture for passive euthanasia. All questions had three options: “yes”, “no,” and “I don’t know”; only affirmative answers were taken as positive. The complete description of the tool can be found in Figure 1.

Group differences were analyzed with the X2 test (SPSS v.25); p-values 0.05-0.10 were considered marginally significant and those <0.05 as significant.

## Results

A total of 1,319 students who explicitly accept their participation in the study were considered for this research. Table 2 shows the frequencies of each question surveyed in the second part of the questionnaire.

**Table 2:** Distribution of frequencies according to the second part of the questionnaire

		Count	Percentage
Gender	Male	598	45.3%
	Female	721	54.7%
Age (groups)	17-22	679	51.5%
	23-25	567	43.0%
	26-30	73	5.5%
Grade	First year	460	34.9%
	Second year	442	33.5%
	Third year	417	31.6%
Religion	Catholic	935	70.9%
	Non-Catholic	185	14.0%
	No religion	199	15.1%
Importance of religious beliefs in daily life	Strong believer	711	53.9%
	Mild believer	473	35.9%
	Non-believer	135	10.2%

Direct posture (questions 1, 7, and 9)

Questions one, seven, and nine addressed the direct posture towards active euthanasia (Q1), passive euthanasia (Q7), and the personal posture towards the topic (Q9). The total frequencies of active (Q1), passive (Q7), and personal posture (Q9) were 44.4% (586), 52.1% (687), and 44.8% (591), respectively.

The Chi-square test was run to find differences between the demographics and direct posture questions (Q1, Q7, and Q9). Table 3 shows the frequencies of positive answers and the significance of each variable.

**Table 3:** Frequency of positive answers for the main questions according to demographics

		Active Euthanasia (Q1)		Passive Euthanasia (Q7)		Personal Posture (Q9)	
		%	P	%	P	%	p
Gender	Male	55.4%	0.001	55.4%	0.031	47.5%	0.740
	Female	49.4%		52.1%		42.6%	
Age	17-22	42.6%	0.359	50.2%	0.000	45.1%	0.168
	23-25	34.6%		55.7%		45.9%	
	25-30	65.8%		41.1%		34.2%	
Grade	First Year	45.9%	0.681	55.7%	0.045	49.6%	0.018
	Second Year	44.6%		47.5%		44.3%	
	Third Year	44.4%		53.0%		40.0%	
Religion	Catholic	41.8%	0.000	51.0%	0.000	43.7%	0.000
	Non-Catholic	34.6%		44.9%		33.0%	
	No religion	65.8%		63.8%		60.8%	
Importance of religious beliefs in daily life	Strong believer	35.0%	0.000	50.6%	0.003	37.7%	0.000
	Mild believer	52.9%		50.3%		50.5%	
	Non-believer	64.4%		65.9%		62.2%	

For the variable gender, significance was meet for question one (p=0.001) and question two (p=0.031). Men tend to have a more positive posture for active and passive euthanasia than women. The variables age and grade weren’t significant and no strong relation could be found in any of the three scenarios.

In contrast to the previous variables, the variables religion and the importance of religion/spirituality in daily life were the ones with major significance (p<0.005 in the six scenarios). For religion (p=0.000), the group that stated no religious beliefs tended to have a more positive posture towards active euthanasia (65.8%), passive euthanasia(63.8%), and personal posture (60.8%), in comparison with the non-Catholic religion group that showed a predominantly negative posture towards the three scenarios. For the variable importance of religious beliefs, the group that stated a strong importance of religion in their daily life had a mainly negative posture towards the topic (35% for active euthanasia, 50.6% for passive euthanasia, 37.7% for personal posture), and the group that considered themselves non-believers had a predominantly positive posture (64.4% for active euthanasia, 65.9% for passive euthanasia, 62.2% for non-believers).

Complementary questions (2-6, 8)

The Chi-square test was run for every variable stated in the second part of the questionnaire versus the six complementary questions to identify if an original negative posture could change in specific scenarios. As with previous groups, significance was found with the variables of religion and the importance of religion/spirituality in daily life. Only relevant findings would be described.

With the variable of religion, although most subgroups keep their original negative answer with all the scenarios present, in only four scenarios did more than 50% of the interviewees change posture. Those cases were: if the actions were legal (Q2) in the non-religious group and in the three subgroups if there were a previously written statement of the patient (Q8) (Table 4).

**Table 4:** Percentages of change from original negative answer to positive answer according to religion

	Religion			
	P	Catholic	Non-Catholic	No Religion
Q2. Would you agree only if the actions were legal?	0.000	44.9%	33.1%	58.8%
Q3. Would you agree even if the actions were illegal?	0.000	1.8%	2.5%	0.0%
Q4. Would you agree to discontinuing therapy, i.e., unplugging a respirator?	0.000	18.2%	24.0%	17.6%
Q5. Would you agree if the physician applied a lethal injection?	0.000	5.3%	7.4%	11.8%
Q6. Would you agree if the physician prescribed a lethal drug?	0.000	5.2%	8.3%	11.8%
Q8. Would you agree only if there is a previous written statement by the patient?	0.000	52.4%	53.9%	68.1%

The same case is true for the variable importance of religion/spiritually in daily life, where four scenarios also showed a change in opinion of about 50%: the groups of mild believer and non-believer in the scenarios of questions two and eight (p=0.000) (Table 5).

**Table 5:** Percentages of change from original negative answer to positive answer according to the importance of religious beliefs/spirituality in daily life

	Importance of religious beliefs/spirituality in daily life			
	P	Strong believer	Mild believer	Non-believer
Q2. Would you agree only if the actions were legal?	0.000	36.8%	56.1%	60.4%
Q3. Would you agree even if the actions were illegal?	0.000	2.4%	0.9%	0.0%
Q4. Would you agree to discontinuing therapy, i.e., unplugging a respirator?	0.000	20.6%	16.6%	16.7%
Q5. Would you agree if the physician applied a lethal injection?	0.000	4.3%	8.5%	10.4%
Q6. Would you agree if the physician prescribed a lethal drug?	0.000	4.1%	9.9%	10.4%
Q8. Would you agree only if there is a previous written statement by the patient?	0.000	48.4%	60.9%	67.4%

Finally, it is important to mention that less than 50% of the interviewees changed their posture in the scenarios propounded in questions three, four, five, and six (p=0.000).

## Discussion

The acceptance of active euthanasia, passive euthanasia, and a favorable personal opinion was 44.4%, 52.1%, and 44.8%, respectively. These results are lower than those seen in previous research made with the same tool in a similar population, where 50% agreed to active euthanasia and 58% to passive euthanasia [6], but they are higher than a previous research applied to physicians, where 40% agreed to active euthanasia and 48% agreed to passive euthanasia [7]. These previous studies suggest the possibility that professional experience or age could be significant factors against passive or active euthanasia, an idea that was previously supported by another study that found that senior doctors tend to have a more negative posture on the topic [9]. However, our study didn't find any significance for the variable age or grade with a positive posture for an active, passive, or personal opinion on the topic. Although our study does not show any difference, it is worth mentioning that the range of age in our population was more than 10 years, the students had no professional experience, and only students from the first three years of medical school were chosen. It is possible that significance could be seen in a higher range of age or in comparison with a population with more years in clinical practice. It is also possible that the significance found with age and clinical experience stated other researches may be due to the different cultural backgrounds seen in older physicians. An in-depth study is needed to clear this interrogative.

For the variable gender, men have a predominantly favorable posture (55.4%) for active (p=0.001) and passive euthanasia (p=0.031), but not for a personal posture (p=0.740). Our findings differ from a previous research, where female residents had a predominantly positive posture to both active and passive euthanasia [6]. We agreed with their discussion that women who are residents may have a profile that differs from an average female student, and that may show the difference in our findings.

However, in our study, the main variables that showed significance were religion and the importance of religion/spirituality in daily life. With religion (p=0.000), the non-Catholic group showed an acceptance lower than 50% for active euthanasia, passive euthanasia, and personal posture versus the group with “no religion,” where the acceptance for the three scenarios was higher than 60%. In a similar case, with “importance of religion/spirituality in daily life” (p<0.0005), the less the spirituality, the stronger the acceptance of euthanasia. These findings agree with previous studies, where physicians who considered themselves atheists had a more positive posture compared to their religion-affiliated counterparts [10-11]. It is worth mentioning that religion is considered the main factor for a negative posture against the topic [7,11]. On the other side, low spirituality is considered one of the main predictors of the pursuit of euthanasia in patients [12].

We further analyze the groups with an initial negative posture who later changed to positive answers in the scenarios described in the complementary questions. The purpose of this analysis was to identify if certain subgroups could change their original posture if the context were different. In our findings, the students who manifest no religion and were mild believers are more prone to change their original negative posture to a positive one if the situation were legal and respected the patient's autonomy (Tables 4-5). These two arguments are two of the most widely discussed to approve euthanasia. The legality of the procedure is considered the main variable to approve euthanasia by physicians [5,9]; however, the “slippery slope” and the dangers of abuse are two common counter-arguments to disagree with the topic [13]. The scenario is similar with the argument for respecting the patient’s autonomy because some clinicians consider autonomy as a major value that needs to be respected, and if the patient wants to limit or ends his life, this idea should be considered without questioning. On the other hand, with a Kantian perspective of autonomy, being autonomous is an unconditionally valuable; that the idea to terminate life is wrong as it terminates this autonomy [14].

## Conclusions

Our study described the current opinion on euthanasia among Mexican medical students from Nuevo Leon, México, relating demographic variables against their personal posture for the topic. Religion and spirituality in daily life were the most important variables to define a positive posture and change an original negative posture to a positive one in a legal context. Although we might never reach a global consensus about euthanasia, it is important to analyze the evolution of thinking about this topic to encourage self-reflection and practice what is best for the patient. Since religion was the most important variable to influence a posture, future studies should focus on this variable and its relevance from the patient’s perspective.
